# Inhibition of miR‐301a‐3p Expression Promotes Ferroptosis: A New Target for Ferroptosis Treatment in TNBC

**DOI:** 10.1155/tbj/9941495

**Published:** 2026-08-28

**Authors:** Baiyang Fu, Yuan Yao, Wenlong Liang, Yao Wang, Xi Chen, Jianguo Zhang

**Affiliations:** ^1^ Department of Breast Surgery, The Second Affiliated Hospital of Harbin Medical University, No. 246 Xuefu Road, Harbin 150000, China, hrbmush.edu.cn; ^2^ Department of Ultrasound, The Second Affiliated Hospital of Harbin Medical University, No. 246 Xuefu Road, Harbin 150000, China, hrbmush.edu.cn

**Keywords:** bioinformatics, ferroptosis, miR-301a-3p, triple-negative breast cancer

## Abstract

**Background:**

The current treatment of triple‐negative breast cancer (TNBC) remains challenging; however, regulating ferroptosis through miRNAs offers new insights for TNBC treatment strategies.

**Methods:**

Bioinformatics methods were used to screen ferroptosis‐related differentially expressed genes (FRDEGs) in TNBC, and ferroptosis‐related miRNAs were screened by combining gene‐miRNA interaction network, miRNA survival analysis, and expression validation in clinical specimens. A nomogram was constructed to evaluate the prognostic impact of miR‐301a‐3p in TNBC. Cell proliferation and migration capabilities were assessed using the CCK‐8 assay, colony formation assay, and wound healing assay, respectively. The intracellular protein expression of GPX4 was measured by Western blot, and fluorescent probes were used to detect intracellular ROS and Fe^2+^ levels.

**Results:**

This study used bioinformatics methods to screen five FRDEGs in TNBC, thereby identifying six core miRNAs that can regulate these genes. Among these, miR‐301a‐3p expression was significantly upregulated in tumor tissues compared to adjacent normal tissues and was associated with poor patient prognosis. Subsequently, an excellent prediction model was established using miR‐301a‐3p expression information and patient clinicopathological information. Functional analyses revealed that miR‐301a‐3p promoted TNBC cell proliferation and migration. In addition, the inhibition of cell proliferation activity by miR‐301a‐3p inhibitor could be reversed by Ferrostatin‐1, and miR‐301a‐3p inhibitor could inhibit the expression of GPX4 and promote the accumulation of intracellular ROS and Fe^2+^. The promotion of cell proliferation activity by miR‐301a‐3p mimic could be reversed by Erastin, and miR‐301a‐3p mimic could promote the expression of GPX4 and inhibit the accumulation of intracellular ROS and Fe^2+^.

**Conclusion:**

In conclusion, this study provides evidence that inhibition of miR‐301a‐3p expression promotes ferroptosis in TNBC cells and produces a synergistic anti‐cancer effect with Erastin, providing a new target for future TNBC ferroptosis treatment strategies.

## 1. Introduction

Breast cancer (BC) is the most common type of cancer in women worldwide [1]. Over the past half‐century, with the development of comprehensive systemic treatments for breast cancer, such as endocrine therapy, chemotherapy, targeted therapy, and immunotherapy, the prognosis of patients has improved [2]. However, triple‐negative breast cancer (TNBC), the most aggressive subtype of BC, often has a poor clinical prognosis due to the lack of clear therapeutic targets and limited treatment options [3]. Although the intensive use of carboplatin [4], capecitabine [5], or olaparib [6] in addition to conventional postoperative chemotherapy can improve the patient’s disease‐free survival (DFS) to a certain extent, the treatment of TNBC is still a difficult problem. Therefore, the development of new therapeutic drugs and treatment strategies remains an important research direction.

Ferroptosis is a nonapoptotic form of cell death. Induction of ferroptosis can inhibit cancer progression, and it has been shown that ferroptosis plays a vital role in TNBC [7]. The molecular mechanism of ferroptosis may be related to the following three points: (1) Iron overload: The imbalance of iron metabolism homeostasis leads to an increase in intracellular Fe^2+^, which plays an important role in ferroptosis [8]. In addition, excess iron can produce reactive oxygen species (ROS) through the Fenton reaction, which further promotes lipid peroxidation [9]. (2) Lipid peroxidation: Intracellular ROS can react with polyunsaturated fatty acids on lipid membranes to generate lipid free radicals and induce lipid peroxidation, leading to the formation of lipid‐ROS (L‐ROS). The further accumulation of L‐ROS leads to ferroptosis [10]. (3) Dysfunction of the antioxidant system: The glutathione peroxidase (GPX) family plays an important protective role in the lipid peroxidation process. Among them, GPX4 can reduce cytotoxic lipid peroxides and play a key role in inhibiting ferroptosis [11].

MicroRNA (miRNA) is a type of short‐chain regulatory RNA that regulates gene expression and influences cellular processes such as growth and death by binding to the 3’ untranslated region (3’ UTR) of target mRNAs [12]. miRNAs perform a wide range of functions in breast cancer, and it has been reported that miRNAs are also involved in the ferroptosis process within breast cancer cells [13]. For instance, both miR‐5096 and miR‐499a‐5p are tumor suppressor miRNAs. Overexpression of miR‐5096 and miR‐499a‐5p can promote ferroptosis and inhibit breast cancer progression [14, 15]. Studies have found that multiple lncRNAs and circRNAs in breast cancer cells can affect the expression of ferroptosis‐related miRNAs. For example, Linc00460, Circ_0000643, CircRHOT1, and CircGFRA1 can act as ceRNAs by competitively binding to ferroptosis‐related miRNAs and reducing the expression of corresponding miRNAs to regulate ferroptosis function [16–19].

In this study, miR‐301a‐3p was identified as a ferroptosis‐related miRNA in TNBC by bioinformatics methods. To investigate the role of miR‐301a‐3p in ferroptosis, MDA‐MB‐231 and BT‐549 cell lines were transfected with miR‐301a‐3p mimic or inhibitor. These results indicate that miR‐301a‐3p is a new potential target for ferroptosis treatment of TNBC.

## 2. Methods

### 2.1. Bioinformatics Analysis

Two gene expression profiles (GSE45827, GSE38959) were retrieved from the GEO database (https://www.ncbi.nlm.nih.gov/geo/), and the sequencing information of TNBC and healthy breast tissues was downloaded. The GEO2R online analysis tool (https://www.ncbi.nlm.nih.gov/geo/geo2r/) and the pheatmap package in R were used to identify differentially expressed genes (DEGs) in TNBC and visualize them, with the filtering conditions set to adj *p* < 0.05 and |log2 (FC)| > 2.

We also obtained a dataset containing 235 genes from the Ferroptosis Database (FerrDb, https://www.zhounan.org/ferrdb/). After excluding noncoding RNAs, we intersected this dataset with the TNBC DEGs to identify ferroptosis‐related differentially expressed genes (FRDEGs) in TNBC. The online tool Hiplot Pro (https://hiplot.com.cn/cloud-tool/drawing-tool/list) was used to generate a Venn diagram.

The gene‐miRNA interaction network was created using NetworkAnalyst (https://www.networkanalyst.ca/). The TarBase v9.0 database was used as the gene‐miRNA interaction database, and the ‘Minimum’ network was displayed in the visualization.

### 2.2. Clinical Data Analysis

A total of 1262 breast cancer samples with complete clinical and gene expression profile data were downloaded from the TCGA database (https://portal.gdc.cancer.gov/), and survival analysis was performed based on miRNA expression levels.

We integrated the clinical characteristic data of TNBC samples in the TCGA database, the expression of miR‐301a‐3p, and the survival information, and established a prognostic nomogram based on multivariate regression to predict the survival prognosis of breast cancer patients. The discrimination ability of the nomogram was quantified by the area under the curve (AUC). The calibration was assessed using calibration curves generated from 1000 bootstrap resamples to evaluate the agreement between the predicted probabilities and actual outcomes.

Clinical tissue specimens were collected from patients who underwent surgery in the Department of Breast Surgery, the Second Affiliated Hospital of Harbin Medical University, between September 2022 and September 2023. Specimens were collected from a total of 10 TNBC patients. All patients were histopathologically diagnosed with TNBC, had no other malignant tumors, and had no major organ diseases. All tissues were snap‐frozen in liquid nitrogen immediately after surgical resection until RNA extraction. This study was approved by the Ethics Committee of the Second Affiliated Hospital of Harbin Medical University, and written informed consent was obtained from all patients.

### 2.3. Cell Culture and Transfection

The human breast cancer cell lines MDA‐MB‐231 and BT‐549 were purchased from iCellbioscience (China), and all cells were cultured in DMEM or RPMI‐1640 medium supplemented with 10% fetal bovine serum and 1% penicillin/streptomycin (both from GIBCO, USA). All cells were incubated at 37°C in a humidified atmosphere containing 5% CO_2_. The miR‐301a‐3p mimic and miR‐301a‐3p inhibitor transfected into cells were both from GenePharma (China). Cell transfections were performed using Lipo8000 transfection reagent (Beyotime, China) following the manufacturer’s protocol.

### 2.4. RNA Extraction and qRT‐PCR

Total RNA was extracted from frozen tissues or cells using TRIzol reagent (Invitrogen, USA) and reverse‐transcribed into cDNA using the miRNA qRT‐PCR Starter Kit (RiboBio, China), and qPCR was performed to determine miRNA expression. U6 was used as a normalization control. All primers are listed in Table 1.

**TABLE 1:** Specific RNAs primers for qRT‐PCR analysis.

Gene	Primer	Sequence (5’−3’)
U6	Forward	ATACAGAGAAAGTTAGCACGG
	Reverse	GGAATGCTTCAAAGAGTTGTG

miR‐27a‐3p	Forward	ATGGTTCGTGGGTTCACA
	Reverse	GTGGCTAAGTTCCGACG

miR‐196a‐5p	Forward	CCGGCTAGGTAGTTTCATGTT
	Reverse	CGGCCCAGTGTTCAGACTAC

miR‐124–3p	Forward	CTCAACTGGTGTCGTGGAGTCGGCAATTCAGTTGAGGGCATTCA
	Reverse	ACACTCCAGCTGGGTAAGGCACGCGGTGAATGCC

miR‐98–5p	Forward	ACACTCCCUAUACAACUUAC
	Reverse	GGGAAAGUAGUGAGGCCTCAGA

miR‐26b‐5p	Forward	GCCACGTTCAAGTAATTCAGGA
	Reverse	CGCAGGGTCCGAGGTATTC

miR‐301a‐3p	Forward	ACACTCCAGCTGGGCAGTGCAATAGTATTGTC
	Reverse	CTCAACTGGTGTCGTGGA

### 2.5. Cell Viability

Cells in 96‐well plates were treated with 5 μmol/L Erastin or 2.5 μmol/L Ferrostatin‐1 for 24 or 48 h. After discarding the drug‐containing medium, 90 μL of medium and 10 μL of CCK‐8 solution (Seven Biotech, China) were added to each well, and the plates were incubated for 2 h. The absorbance was measured at 450 nm using a microplate reader (Bio‐Rad, USA).

### 2.6. Colony Formation Assays

Approximately 1000 cells were seeded in a 6‐well plate and cultured in DMEM or RPMI‐1640 complete medium. After 2 weeks, the cells were washed with PBS, fixed in methanol for 30 min, stained with 1% crystal violet dye, photographed, and counted.

### 2.7. Wound Healing Assay

Approximately 5 × 10^5^ cells were seeded in a 6‐well plate and incubated overnight so that the 6‐well plate was covered with cells in a monolayer. A straight line was drawn in the well using a 20 μL pipette tip. The wells were then washed three times with PBS, and the medium was replaced with serum‐free medium for continued culture. Pictures were taken at appropriate time points, and the cell migration area was calculated.

### 2.8. Western Blot

Cell samples were collected and lysed with protein extraction buffer. Proteins were separated by SDS‐PAGE and subsequently transferred onto PVDF membranes (Millipore, USA). After blocking with 5% skim milk powder, the membranes were incubated with primary antibodies overnight at 4°C and with secondary antibodies for 2 h at room temperature. Chemiluminescent detection reagents (Beyotime, China) were used for exposure and development. The experimental results were analyzed and quantified using image analysis software.

### 2.9. Intracellular ROS Analysis

Intracellular ROS were detected using DCFH‐DA (Beyotime, China). Following the manufacturer’s instructions, cells in 6‐well plates were treated with either 5 μmol/L Erastin or 2.5 μmol/L Ferrostatin‐1 for 24 h. After discarding the drug‐containing medium, the cells were co‐incubated with the 10 μM DCFH‐DA probe at 37°C for 30 min. After removing the DCFH‐DA, the cells were washed three times with HBSS buffer. Fluorescence images were acquired using a fluorescence microscope (Nikon, Japan).

### 2.10. Intracellular Fe^2+^ Analysis

Intracellular Fe^2+^ was detected by FerroOrange (Dojindo, Japan). Following the manufacturer’s instructions, cells in 6‐well plates were treated with either 5 μmol/L Erastin or 2.5 μmol/L Ferrostatin‐1 for 24 h. After discarding the drug‐containing medium, the cells were washed three times with HBSS, and then the cells were incubated with 1 μM FerroOrange at 37°C for 30 min. Fluorescence images were captured using a fluorescence microscope.

### 2.11. Statistical Analysis

All statistical analyses were performed using R software (Version 4.1.0) and GraphPad Prism (version 9.5). All experiments were independently repeated at least three times. Normality was assessed using the Shapiro–Wilk test before applying parametric tests. For data that followed a normal distribution, Student’s *t*‐test was used for comparisons between two groups, and one‐way analysis of variance was used for comparisons among multiple groups. For data that did not follow a normal distribution, nonparametric tests were applied, including the Mann–Whitney *U* test for comparisons between two groups and the Kruskal–Wallis test for comparisons among multiple groups. *p* < 0.05 was considered statistically significant (ns, not significant; ^∗^, *p* < 0.05; ^∗∗^, *p* < 0.01; ^∗∗∗^, *p* < 0.001; ^∗∗∗∗^, *p* < 0.0001).

## 3. Results

### 3.1. Screening Out Five FRDEGs in TNBC

First, to identify DEGs in TNBC, we set |log2 (FC)| > 2 and adj *p* < 0.05 as the screening thresholds. A total of 1736 DEGs were identified in the GSE45827 dataset. The results were visualized using heat maps and volcano plots (Figure 1A,C). In the GSE38959 dataset, 673 DEGs were identified (Figure 1B,D). Subsequently, a Venn diagram was used to determine the intersection results of the above two DEG gene sets and the 235 ferroptosis‐related genes in the FerrDb gene set (Figure 1E). Finally, five FRDEGs were obtained, including IDO1, CDO1, SAT1, LIFR, and ACADSB.

Figure 1(A, B) Heat maps of DEGs between normal and TNBC groups (GSE45827 and GSE38959). DEGs were screened using |log2 (FC)| > 2 and adj *p* < 0.05. (C, D) Volcano maps of DEGs between normal and TNBC groups (GSE45827 and GSE38959). (E) Venn diagram showing FRDEGs.

**Figure:**
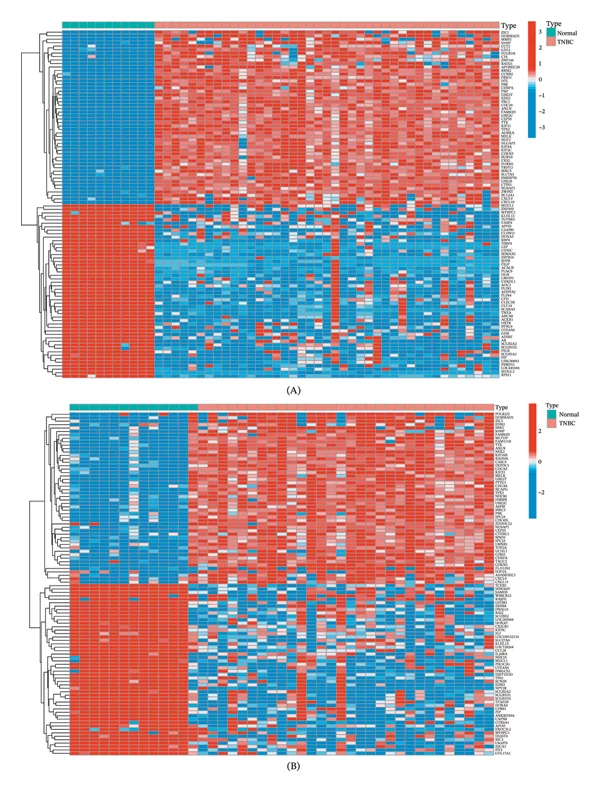

**Figure:**
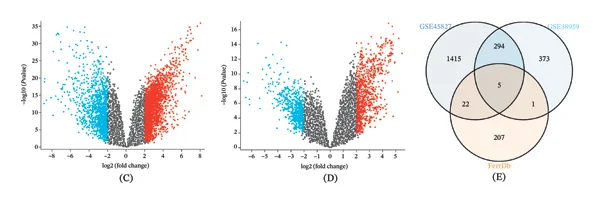

### 3.2. Screening and Validation of Ferroptosis‐Related miRNAs in TNBC

**Figure 2:**
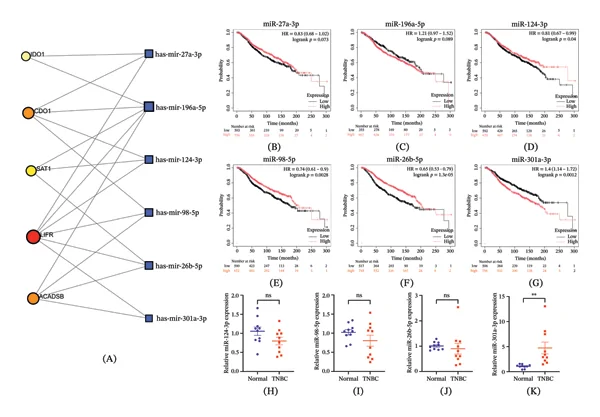
(A) mRNA‐miRNA interaction network of FRDEGs. (B–G) Kaplan–Meier survival curves of patients with high and low expression of miR‐27a‐3p, miR‐196a‐5p, miR‐124‐3p, miR‐98‐5p, miR‐26b‐5p, and miR‐301a‐3p. Survival differences were assessed using the log‐rank test, and the hazard ratio (HR) with 95% confidence interval (CI) was used as the effect‐size estimate. Exact *p* values and subgroup *n* values are shown in the corresponding panels. (H–K) Differential expression of miR‐124‐3p, miR‐98‐5p, miR‐26b‐5p, and miR‐301a‐3p between tumor and adjacent tissues of TNBC patients (*n* = 10). Data are presented as mean ± SD. *p* < 0.05 was considered statistically significant (ns, not significant; ^∗^ *p* < 0.05; ^∗∗^ *p* < 0.01; ^∗∗∗^ *p* < 0.001; ^∗∗∗∗^ *p* < 0.0001).

To identify ferroptosis‐related miRNAs, we first generated an interaction network between FRDEGs and miRNAs through network analysis. The “Minimu” network setting was applied to display miRNAs that interacted with a relatively large number of genes (≥ 2) (Figure 2A). A total of six potential ferroptosis‐related miRNAs (miR‐27a‐3p, miR‐196a‐5p, miR‐124‐3p, miR‐98‐5p, miR‐26b‐5p, and miR‐301a‐3p) were identified. These miRNAs were found to regulate multiple FRDEGs, indicating that they play a core role in regulation. To further determine the prognostic effects of these miRNAs on breast cancer patients, we analyzed the survival of 1262 breast cancer cases in the TCGA database according to the expression of miRNAs. Survival analysis showed that there was no significant difference in survival between patients with low and high expression of miR‐27a‐3p and miR‐196a‐5p (*p* > 0.05) (Figure 2B,C). Higher expression levels of miR‐124‐3p, miR‐98‐5p, and miR‐26b‐5p were associated with a better survival rate (*p* < 0.05) (Figure 2D–F). Conversely, high expression of miR‐301a‐3p was associated with a poorer prognosis (*p* < 0.05) (Figure 2G). We then focused on miR‐124‐3p, miR‐98‐5p, miR‐26b‐5p, and miR‐301a‐3p, four miRNAs associated with survival differences, and performed qRT‐PCR analysis of miRNA expression in 10 primary TNBC tissue samples and matched adjacent breast tissues. We found that only miR‐301a‐3p was differentially expressed in TNBC tissues compared with adjacent tissues (*p* < 0.01), and the level of miR‐301a‐3p in TNBC was significantly increased (Figure 2H–K). These findings suggest that miR‐301a‐3p is a potential ferroptosis‐related miRNA in TNBC.

### 3.3. Construction and Validation of a Nomogram for Predicting OS in TNBC Patients Based on miR‐301a‐3p Expression and Clinical Information

**Figure 3:**
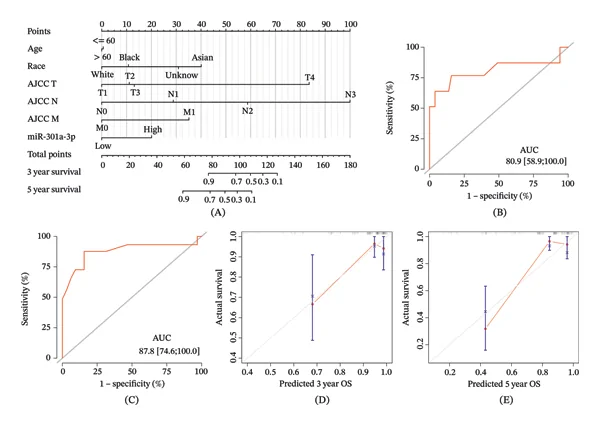
(A) Nomogram combining miR‐301a‐3p expression and clinical information. (B, C) ROC curves for 3 year and 5 year survival rates. The 3 year and 5 year AUC values were 0.809 and 0.878, respectively. (D, E) Calibration curves for 3‐ and 5 year survival probabilities generated using 1000 bootstrap resamples.

To further explore the risk level and prognostic effect of miR‐301a‐3p on TNBC patients, we combined the miR‐301a‐3p expression and clinical characteristics of TNBC patients in the TCGA database to establish a nomogram for predicting the 3‐year and 5‐year survival rates of TNBC patients (Figure 3A). The total score obtained by adding the corresponding scores of each clinical variable and miR‐301a‐3p expression of the patient can predict the patient’s prognosis. The higher the total score, the worse the patient’s prognosis. To verify the accuracy of the model in predicting prognosis, we also plotted the ROC curve and calibration curve of the 3 year and 5 year survival rates of TNBC patients. The 3 year and 5 year nomogram AUCs were 0.809 and 0.878, respectively (Figure 3B,C). The calibration curves demonstrated good agreement between the predicted and observed outcomes (Figure 3D,E). Excellent discrimination and calibration demonstrated the accuracy of the prognostic model.

### 3.4. miR‐301a‐3p Can Regulate the Proliferation and Migration of TNBC Cells

**Figure 4:**
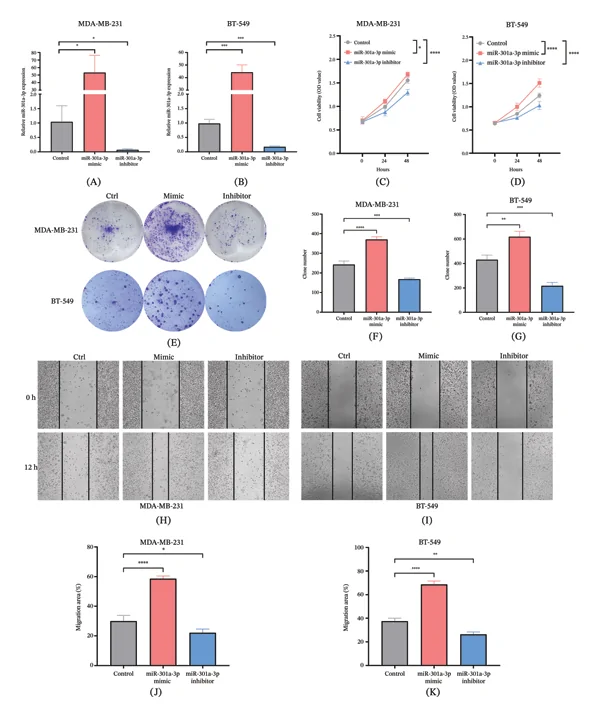
(A, B) Relative expression of miR‐301a‐3p detected by qRT‐PCR. (C, D) OD values of each group were detected by the CCK‐8 assay. (E–G) Colony formation assay to detect the number of clones formed in different treatment groups. (H–K) The migration area of cells in each group was detected by the wound healing assay. Data are presented as mean ± SD. In vitro experiments were repeated at least three times. *p* < 0.05 was considered statistically significant (ns, not significant; ^∗^, *p* < 0.05; ^∗∗^ *p* < 0.01; ^∗∗∗^ *p* < 0.001; ^∗∗∗∗^ *p* < 0.0001).

To investigate the functional role of miR‐301a‐3p in vitro, we utilized two human TNBC cell lines, MDA‐MB‐231 and BT‐549. Next, we transfected MDA‐MB‐231 and BT‐549 cells with miR‐301a‐3p mimic or inhibitor into MDA‐MB‐231 and BT‐549 cells, respectively, and verified the transfection efficiency by qRT‐PCR. The results showed that we successfully constructed the miR‐301a‐3p mimic group and miR‐301a‐3p inhibitor group of MDA‐MB‐231 and BT‐549 cells (Figure 4A,B), and the expression difference was statistically significant. To assess the impact of miR‐301a‐3p on cellular phenotypes, we performed CCK‐8 assays, colony formation assays, and wound healing assays to evaluate its effects on the proliferation and migration capabilities of TNBC cells. We measured the cell activity of different treatment groups at 0, 24, and 48 h using the CCK‐8 assay (Figure 4C,D). The results showed that after 48 h of culture, the proliferation activity of the miR‐301a‐3p mimic group was higher than that of the control group, and the proliferation activity of the miR‐301a‐3p inhibitor group was significantly lower than that of the control group. We further performed colony formation assays (Figure 4E–G). Compared with the control group, the number of colonies in the miR‐301a‐3p mimic group increased significantly, and the number of colonies in the miR‐301a‐3p inhibitor group decreased significantly, and the above results were statistically significant. Furthermore, the wound healing assay results (Figure 4H–K) indicated that miR‐301a‐3p mimic significantly enhanced the migration ability of TNBC cells, and miR‐301a‐3p inhibitor inhibited the migration ability of TNBC cells.

### 3.5. Low Expression of miR‐301a‐3p Promotes Ferroptosis of TNBC Cells

The results of CCK‐8 experiments showed that the proliferation activity of the miR‐301a‐3p inhibitor group was lower than that of the control group, but the addition of the ferroptosis inhibitor Ferrostatin‐1 significantly offset the changes in cell proliferation activity associated with the miR‐301a‐3p inhibitor, indicating that ferroptosis was involved (Figure 5A,B). Furthermore, the miR‐301a‐3p inhibitor enhanced the suppression of cell growth mediated by the ferroptosis inducer Erastin, suggesting a synergistic effect (Figure 5C,D). In addition, we examined GPX4, a key indicator of ferroptosis defense capabilities. Western blot results (Figure 5E–H) showed that GPX4 protein expression was lower in the miR‐301a‐3p inhibitor group than in the control group. Next, we evaluated the level of ferroptosis by measuring the intracellular ROS and Fe^2+^ levels (Figure 5I–P). miR‐301a‐3p inhibitor significantly increased the intracellular ROS and Fe^2+^ levels, and miR‐301a‐3p inhibitor promoted the increase in intracellular ROS and Fe^2+^ levels induced by Erastin. In contrast, the increased fluorescence intensity of ROS and Fe^2+^ in cells transfected with miR‐301a‐3p inhibitor could be reversed by Ferrostatin‐1. Collectively, these findings demonstrate that inhibition of miR‐301a‐3p promotes ferroptosis in TNBC cells and synergizes with Erastin to enhance ferroptosis.

Figure 5(A–D) CCK‐8 assay to detect the OD value of miR‐301a‐3p low‐expressing cells treated with Erastin (5 μmol/L) and Ferrostatin‐1 (2.5 μmol/L). (E–H) Western blot assay to detect the effect of low miR‐301a‐3p expression on GPX4 protein expression level. (I, J, M, N) DCFH‐DA probe‐treated cells to detect intracellular ROS (scale bar, 100 μm). (K, L, O, P) FerroOrange probe‐treated cells to detect intracellular Fe^2+^ (scale bar, 100 μm). Data are presented as mean ± SD. In vitro experiments were repeated at least three times. *p* < 0.05 was considered statistically significant (ns, not significant; ^∗^ *p* < 0.05; ^∗∗^ *p* < 0.01; ^∗∗∗^ *p* < 0.001; ^∗∗∗∗^ *p* < 0.0001).

**Figure:**
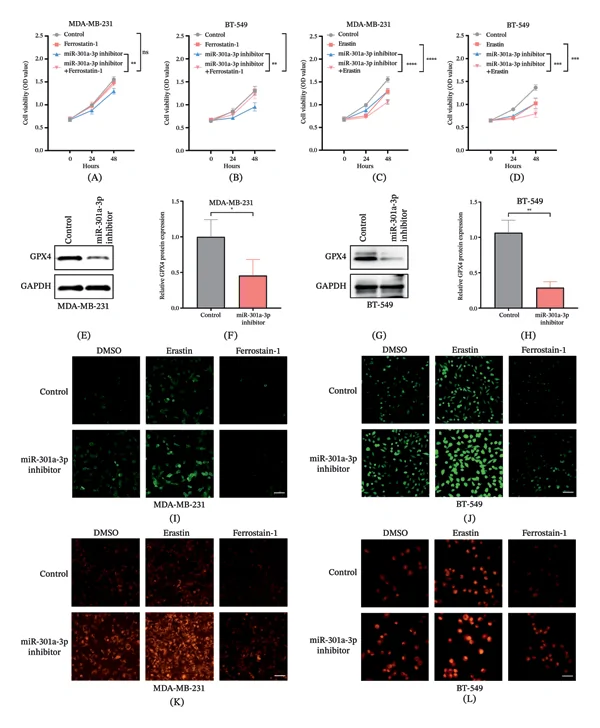

**Figure:**
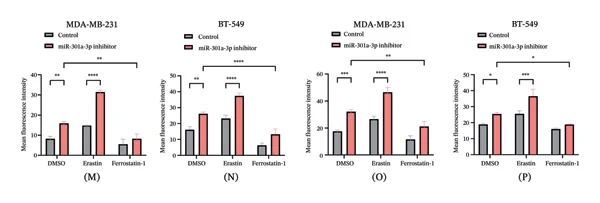

### 3.6. Overexpression of miR‐301a‐3p Inhibits Ferroptosis of TNBC Cells

CCK‐8 results showed that the cell proliferation activity of the miR‐301a‐3p mimic + Erastin group was higher than that of the Erastin group, but the proliferation activity of the miR‐301a‐3p mimic + Ferrostatin‐1 group was no different from that of the Ferrostatin‐1 group (Figure 6A–D). Western blot results (Figures 6E–H) showed that GPX4 protein expression was higher in the miR‐301a‐3p mimic group than in the control group. These findings suggest that miR‐301a‐3p enhanced the ferroptosis defense capacity of TNBC cells. In addition, miR‐301a‐3p mimic significantly reduced the levels of ROS and Fe^2+^ in cells, and even in the presence of Erastin, miR‐301a‐3p mimic could still reduce the fluorescence intensity of ROS and Fe^2+^ (Figure 6I–P). These results indicate that miR‐301a‐3p mimic can inhibit ferroptosis in TNBC cells and can inhibit ferroptosis induced by Erastin.

Figure 6(A–D) CCK‐8 assay to detect the OD value of miR‐301a‐3p overexpressing cells treated with Erastin (5 μmol/L) and Ferrostatin‐1 (2.5 μmol/L). (E–H) Western blot assay to detect the effect of miR‐301a‐3p overexpression on GPX4 protein expression level. (I, J, M, N) DCFH‐DA probe‐treated cells to detect intracellular ROS (scale bar, 100 μm). (K, L, O, P) FerroOrange probe‐treated cells to detect intracellular Fe^2+^ (scale bar, 100 μm). Data are presented as mean ± SD. In vitro experiments were repeated at least three times. *p* < 0.05 was considered statistically significant (ns, not significant; ^∗^ *p* < 0.05; ^∗∗^ *p* < 0.01; ^∗∗∗^ *p* < 0.001; ^∗∗∗∗^ *p* < 0.0001).

**Figure:**
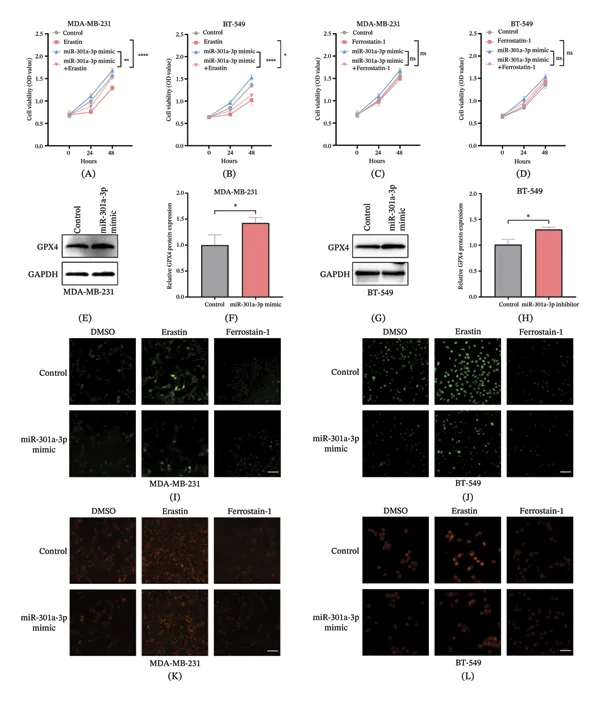

**Figure:**
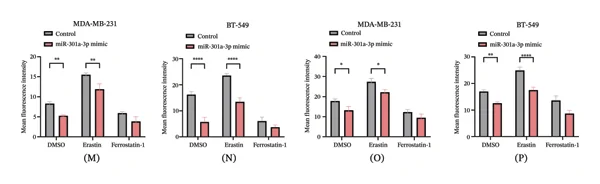

## 4. Discussion

In 2020, the number of new breast cancer cases worldwide accounted for 11.7% of all cancers, surpassing lung cancer to become the most frequently diagnosed cancer [1]. TNBC accounts for 15%–20% of all breast cancers and is characterized by high malignancy, high recurrence and metastasis rates, and poor prognosis [20]. As a classic tumor regulatory molecule, miR‐301a‐3p plays a vital role in the occurrence and development of breast cancer and is also considered a potential therapeutic target [21]. However, the effect of miR‐301a‐3p on ferroptosis in TNBC cells had not been reported before this study. In this study, our findings suggest that inhibition of miR‐301a‐3p expression promotes ferroptosis in TNBC cells, supporting miR‐301a‐3p as a potential therapeutic target for ferroptosis‐based strategies.

Unlike other forms of death, such as apoptosis and pyroptosis, ferroptosis is characterized by iron accumulation, lipid peroxidation, and imbalance of the antioxidant system and is a form of cell death that destroys the integrity of biological membranes [22]. GPX4 and SLC7A11 are key negative regulators of ferroptosis. GPX4 is a glutathione (GSH)‐dependent peroxidase that can convert GSH into oxidized glutathione (GSSG) and reduce cytotoxic lipid peroxides to the corresponding alcohols. SLC7A11 is a subunit of the cystine/glutamate antiporter (System Xc‐), which is responsible for the cell’s uptake of cystine, excretion of glutamate, and synthesis of GSH [23]. Studies have shown that a variety of miRNAs can play a role in the ferroptosis process of cancer. For example, miR‐5096 inhibits the transport of cystine into cells by targeting and downregulating SLC7A11, thereby increasing intracellular ROS and ultimately promoting ferroptosis of TNBC cells. In addition, miR‐5096 can also play the role of a tumor suppressor miRNA by inhibiting the colony formation, migration, invasion, and epithelial‐mesenchymal transition of TNBC cells [15]. The tumor suppressor miRNA miR‐106a‐5p targets and inhibits STAT3 to increase the levels of intracellular ROS and Fe^2+^ and reduce the levels of GPX4 and SLC7A11 proteins, ultimately promoting the occurrence of ferroptosis and inhibiting cancer progression [19]. Ferroptosis plays an important role in breast cancer. Deciphering the mechanism of ferroptosis, discovering new targeted drugs, or finding drugs that can synergistically enhance the sensitivity of existing drugs can provide new treatment strategies for TNBC [24].

In this study, we identified five FRDEGs in TNBC using bioinformatics. Based on these findings, an mRNA‐miRNA interaction network was constructed to identify six core miRNAs capable of regulating multiple FRDEGs. Next, through survival difference analysis and clinical specimen verification, it was found that five of the six candidate miRNAs did not affect the survival of patients or had no expression differences in clinical tissue specimens. Only miR‐301a‐3p was expressed at higher levels in tumor tissues compared to normal tissues, and its elevated expression in BC tissues was associated with a poorer prognosis. This indicates that miR‐301a‐3p can not only affect the malignancy of TNBC but also play an important role in the ferroptosis process of TNBC.

Previous research data showed that miR‐301a‐3p, as a cancer‐promoting miRNA, is highly expressed in a variety of tumor cells and tissues, such as esophageal cancer [25], prostate cancer [26], and colorectal cancer [27]. This indicates that it is closely related to the occurrence of a variety of cancers. At the same time, because miR‐301a‐3p can function by targeting a variety of mRNAs, it affects the proliferation, migration, and invasion of tumors, thereby changing the prognosis of patients. Studies have shown that miR‐301a‐3p can negatively regulate Smad4 to promote the proliferation, migration, and invasion of pancreatic cancer and laryngeal cancer cells, and high expression of miR‐301a‐3p represents a poor prognosis [28, 29]. In liver cancer, miR‐301a‐3p is highly expressed in tissues and cells and indicates a poor prognosis. At the same time, miR‐301a‐3p can target VGLL4 to enhance the proliferation and invasion of liver cancer cells [30].

In this study, we established groups of cells transfected with miR‐301a‐3p mimic or inhibitor. The results demonstrated that miR‐301a‐3p overexpression promoted the proliferation, colony formation, and wound healing capacity of TNBC cells, whereas its knockdown yielded the opposite effects. As a cancer‐promoting miRNA, the results of this study can prove that the effects of miR‐301a‐3p on the proliferation and migration of TNBC cells are consistent with its performance in other tumors. In addition, the ferroptosis inhibitor Ferrostatin‐1 reversed the miR‐301a‐3p inhibitor‐mediated attenuation of TNBC cell proliferation activity, indicating that ferroptosis may be involved in the change of proliferation activity. Further experimental results showed that the miR‐301a‐3p inhibitor inhibited the expression of GPX4 and promoted the accumulation of intracellular ROS and Fe^2+^, which confirmed the occurrence of ferroptosis. Moreover, when miR‐301a‐3p inhibitor and Erastin acted together on TNBC cells, their proliferation activity was further inhibited, and intracellular ROS and Fe^2+^ accumulated further, which proved that miR‐301a‐3p inhibitor and Erastin synergized to enhance the ferroptosis effect on cells. While miR‐301a‐3p mimic inhibited Erastin‐induced ferroptosis, miR‐301a‐3p mimic promoted GPX4 expression and inhibited the accumulation of intracellular ROS and Fe^2+^, which proved that miR‐301a‐3p overexpression can inhibit ferroptosis of TNBC cells. To the best of our knowledge, few studies have linked miR‐301a‐3p to ferroptosis regulation in TNBC. Our findings add to emerging evidence that inhibition of an oncomiR may promote ferroptosis in cancer cells. This finding is consistent with reports in other cancer types that support our conclusion. For example, in gastric cancer, inhibition of the cancer‐promoting miRNA miR‐522 can increase ROS in gastric cancer cells, thereby promoting ferroptosis of gastric cancer cells [31]. In pancreatic cancer, inhibition of the cancer‐promoting miRNA miR‐3173‐5p can increase ACSL4 levels, thereby promoting the accumulation of intracellular ROS and Fe^2+^, ultimately promoting pancreatic cancer ferroptosis and attenuating gemcitabine resistance in pancreatic cancer [32].

The present study still has several limitations. First, the number of clinical tissue samples used for qRT‐PCR validation was relatively limited, and all samples were obtained from a single center. This may limit the generalizability of the tissue‐level validation results. In addition, heterogeneity in tumor stage, treatment status, and other clinicopathological characteristics among TNBC patients may also affect miR‐301a‐3p expression levels. Therefore, future studies should further validate the expression characteristics and clinical significance of miR‐301a‐3p in larger, multicenter TNBC cohorts with more detailed clinicopathological stratification. Second, the nomogram constructed in this study was mainly based on TCGA data and was internally evaluated using ROC curves and calibration curves. However, validation in an independent external cohort is still lacking. Therefore, the clinical applicability and predictive stability of this model need to be further confirmed in external TNBC cohorts. Third, the direct mRNA targets through which miR‐301a‐3p regulates ferroptosis remain to be fully defined. Although GPX4 was used as a ferroptosis‐defense marker in this study, the current evidence does not establish GPX4 as a direct target of miR‐301a‐3p. Further experimental studies are needed to clarify the downstream targets and molecular mechanisms involved in miR‐301a‐3p‐mediated ferroptosis regulation. In summary, our results confirm that miR‐301a‐3p is a cancer‐promoting miRNA, whose high expression can promote the proliferation and migration of TNBC cells and is associated with a poorer prognosis. In addition, we found that inhibiting miR‐301a‐3p expression can promote TNBC cell ferroptosis and produce a synergistic anticancer effect with Erastin, providing a new potential target for future TNBC ferroptosis treatment strategies.

## 5. Conclusion

This study suggests that miR‐301a‐3p is a ferroptosis‐related miRNA in TNBC. Inhibition of miR‐301a‐3p expression can significantly promote ferroptosis in TNBC cells by reducing GPX4 levels and increasing the accumulation of intracellular ROS and Fe^2+^. At the same time, our experiments showed that combined treatment with Erastin and a miR‐301a‐3p inhibitor can produce a synergistic effect and significantly enhance ferroptosis‐mediated anticancer effects. This study provides a new perspective for ferroptosis‐based targeted treatment of TNBC. In the future, the development of inhibitors targeting miR‐301a‐3p is expected to become a new strategy to improve the therapeutic effect of TNBC and also lay the foundation for the precise treatment of combined ferroptosis inducers. Further clinical studies will help verify the feasibility and effectiveness of miR‐301a‐3p targeted therapy, thereby promoting the development of personalized treatment for TNBC.

**Nomenclature**
BC: Breast cancer
TNBC: Triple‐negative breast cancer
DFS: Disease‐free survival
ROS: Reactive oxygen species
GPX: Glutathione peroxidase
miRNA: MicroRNA
FRDEG: Ferroptosis‐related differentially expressed gene
AUC: Area under curve
DEGs: Differentially expressed genes

## Author Contributions

Baiyang Fu: conceptualization, methodology, formal analysis, investigation, data curation, and writing–original draft. Yuan Yao: methodology, formal analysis, investigation, data curation, and writing–review and editing. Wenlong Liang: investigation, data curation, and validation. Yao Wang: investigation, data curation, and validation. Xi Chen: conceptualization, supervision, and writing–review and editing. Jianguo Zhang: conceptualization, supervision, funding acquisition, and writing–review and editing. Baiyang Fu and Yuan Yao contributed equally to this work.

## Funding

This work was supported by the National Natural Science Foundation of China (No. 81972469) and the Graduate Practical Innovation Project of Harbin Medical University (No. YJSCX2025‐58HYD).

## Disclosure

All authors have read and approved the final version of the manuscript. The corresponding author, Jianguo Zhang, had full access to all of the data in this study and takes complete responsibility for the integrity of the data and the accuracy of the data analysis.

The corresponding author and manuscript guarantor, Jianguo Zhang, hereby affirms that this manuscript represents an honest, accurate, and transparent account of the study being reported. No important aspects of the study have been omitted, and any discrepancies from the study as originally planned have been explicitly explained.

## Ethics Statement

Informed consent was provided by all patients, and the Ethics Committee of The Second Affiliated Hospital of Harbin Medical University approved all aspects of this study.

## Conflicts of Interest

The authors declare no conflicts of interest.

## Data Availability

The publicly available datasets analyzed in this study were obtained from the GEO, FerrDb, and TCGA databases. The data that support the findings of this study are available on request from the corresponding authors.
